# Application of Finite Element Analysis in Oral and Maxillofacial Surgery—A Literature Review

**DOI:** 10.3390/ma13143063

**Published:** 2020-07-09

**Authors:** Magdalena Lisiak-Myszke, Dawid Marciniak, Marek Bieliński, Hanna Sobczak, Łukasz Garbacewicz, Barbara Drogoszewska

**Affiliations:** 1 Maxillofacial Surgery Ward, Alfa-Med Medical Center, 85-095 Bydgoszcz, Poland; 2 Faculty of Mechanical Engineering, Department of Manufacturing Technology, UTP University of Science and Technology, 85-796 Bydgoszcz, Poland; dawid.marciniak@utp.edu.pl (D.M.); biel@utp.edu.pl (M.B.); 3 Department of Maxillofacial Surgery, Medical University of Gdansk, 80-210 Gdansk, Poland; hanna.sobczak@gumed.edu.pl (H.S.); lukasz.garbacewicz@gumed.edu.pl (Ł.G.); barbara.drogoszewska@gumed.edu.pl (B.D.)

**Keywords:** finite element analysis, FEA, oral surgery, maxillofacial surgery

## Abstract

In recent years in the field of biomechanics, the intensive development of various experimental methods has been observed. The implementation of virtual studies that for a long time have been successfully used in technical sciences also represents a new trend in dental engineering. Among these methods, finite element analysis (FEA) deserves special attention. FEA is a method used to analyze stresses and strains in complex mechanical systems. It enables the mathematical conversion and analysis of mechanical properties of a geometric object. Since the mechanical properties of the human skeleton cannot be examined in vivo, a discipline in which FEA has found particular application is oral and maxillofacial surgery. In this review we summarize the application of FEA in particular oral and maxillofacial fields such as traumatology, orthognathic surgery, reconstructive surgery and implantology presented in the current literature. Based on the available literature, we discuss the methodology and results of research where FEA has been used to understand the pathomechanism of fractures, identify optimal osteosynthesis methods, plan reconstructive operations and design intraosseous implants or osteosynthesis elements. As well as indicating the benefits of FEA in mechanical parameter analysis, we also point out the assumptions and simplifications that are commonly used. The understanding of FEA’s opportunities and advantages as well as its limitations and main flaws is crucial to fully exploit its potential.

## 1. Introduction

**Figure 1:**
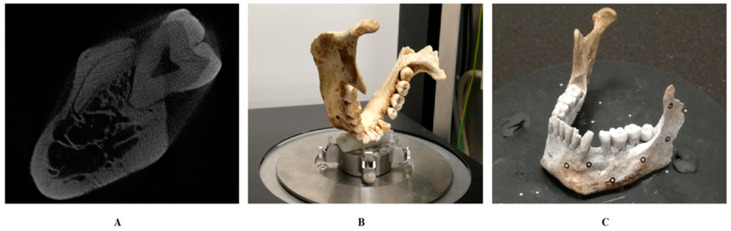
Different methods of obtaining a three-dimensional image of a human mandible. (**A**) Human mandible cone beam tomography (CBCT) cross-section. (**B**) Human mandible prepared for microtomography. (**C**) Human mandible prepared for extraoral scanning.

**Figure 2:**
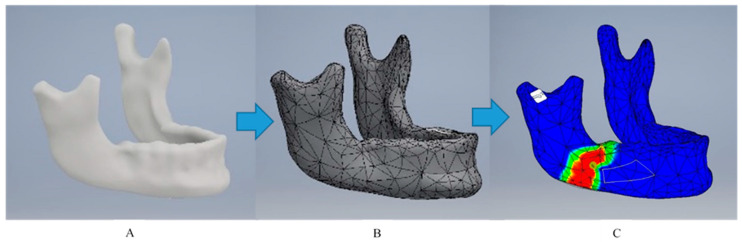
Obtaining a numerical model (**A**) is followed by its division into many simple finite elements that are connected at a common nodal point (discretization) (**B**). After each element is assigned appropriate material properties, the finite element analysis (FEA) of simulated forces at different regions of the numerical model can be conducted and the distributions of specific stresses and strains revealed (**C**).

Finite element analysis is an advanced numerical method for conducting computer-aided engineering. From a mathematical perspective it enables obtaining approximate solutions to the partial differential equations that constitute a mathematical model for a given process or status of a physical system. In terms of its practical application, FEA is commonly used by various researchers to analyze stresses and strains in complex mechanical systems [1]. The implementation of FEA in solving research problems requires certain steps. At the first stage, it is essential to create a numerical model of the object which is to be tested. In dentistry, a geometric model which constitutes a basis for its virtual digital equivalent is typically a three-dimensional image of a part of the human stomatognathic system obtained using cone beam computed tomography (CBCT), microtomography, intra- and extraoral scanners (Figure 1) or computer-aided design (CAD) software. The creation of a numerical model is followed by its discretization, which means its division into many simple elements (finite elements) that are connected at a common nodal point. Each element is assigned appropriate material properties, such as Young’s modulus (E) or Poisson’s ratio (v) of the bone, teeth or foreign body, which were previously determined during the course of the experimental studies [2]. Finally, the application of simulated forces at different regions of the numerical model can be conducted and the distributions of specific stresses and strains revealed (Figure 2).

The area of dentistry which has always been tightly associated with biomechanics is oral and maxillofacial surgery. The progress of each of its subfields, such as trauma surgery, orthognathic surgery, reconstructive surgery and implantology, is strictly connected with the understanding of the pathomechanism of fractures, the biological responses of bone to biomechanical changes and the behavior of osteosynthesis materials and intraosseous implants, in both healthy and pathological conditions.

Oral and maxillofacial pathologies have always been present, and so has the need to search for their optimal treatment methods. In the field of maxillofacial reconstructive surgery, the glass eyes, metal noses and ivory teeth discovered on Egyptian mummies represent a good example. In the matter of handling facial trauma, methods of immobilizing broken bones have been a subject of interest for many surgeons. Hippocrates studied the intraoral fixation of a dislocated mandible by using a golden thread tied around the teeth adjacent to the fracture site. Over time, Ivy, Salicetti, Gilmer and Gunning searched for other techniques of maxillomandibular fixation in the design of different wires and splints [3], until between 1973 and 1975 Michelet, Champy and Lodde introduced the use of miniplate osteosynthesis which nowadays stands as the golden standard [4]. Without modern study aids, the materials implemented initially in maxillofacial surgery did not immediately meet the anticipated expectations, undergoing fractures and causing the displacement of fracture segments, discomfort and healing complications [5]. Thus, the demand for development in biomechanical and biomaterial science emerged. In the early twentieth century, Sicher and Tandler introduced the concepts of structural pillars which represented the areas of the skull most resistant to mechanical induction [6] and René Le Fort investigated human skulls in order to understand the pathomechanism of facial injuries [7].

Nowadays, owing to the advancement of computing, we lean towards virtual analysis and tools complying to our needs for testing the mechanical properties of facial hard and soft tissues, osteosynthesis materials, implant components and different biological and synthetic bone substitute biomaterials, regardless of their complexity, in a more accurate, repetitive, safe and cost-effective way [8]. FEA corresponds to that need [9,10]. Thanks to FEA, models which give feedback on the biological responses of bone to biomechanical changes can be easily generated and numerous complementary components to determine the impact on the tested subject as well as the adjacent anatomical elements can be simulated. It also enables the testing of different fixation systems before applying them to patients, allowing the prevention of future failure derived from its unsuitable selection or positioning.

The maxillofacial region contains many essential anatomical structures. Interventions in this area require precise, well-arranged cutting lines, maintaining or restoring the functionality of tissues and obtaining predictable long-term mechanical outcomes. FEA, as the methodology which allows us to conduct different biomechanical simulations in the complex maxillofacial anatomy, while remaining repeatable, timesaving and cost-effective at the same time, is a highly valuable research tool.

In the current literature a lack of updated complex coverage of the discussed subject is noticeable. The purpose of this article is to present in a maximally comprehensive way the ways in which FEA has already contributed to research in the specific areas of oral and maxillofacial surgery such as trauma surgery, orthognathic surgery, reconstructive surgery and implantology and to outline EFA’s present limitations as well as its forthcoming possibilities. It organizes the information on FEA’s usability and promotes the wider implementation of numerical methods in oral and maxillofacial studies.

## 2. Materials and Methods

Based on contemporary articles and reports printed in reputable journals the review of the literature available on the featured subject was undertaken. The research was conducted using PubMed, Medline, Google Scholar, and the available books and reports. The key words “finite element study”, “finite element analysis”, “finite element modeling”, “oral surgery”, “maxillofacial surgery”, “traumatology”, “orthognathic surgery”, “reconstructive surgery”, “implantology” and a combination of thereof were used to select the literature form the databases. Afterwards, given the large amount of information available on this topic, the authors divided the accessed papers into groups, assigning them to the principal fields of oral and maxillofacial surgery such as trauma surgery, orthognathic surgery, reconstructive surgery and implantology, selecting a representative sample for each group. In order to preserve clarity, they are discussed in sections corresponding to the categories indicated above.

## 3. Trauma Surgery

Investigating facial trauma’s consequences and etiology in vivo is practically impossible because of ethical reasons. The creation of a reliable physical test model which satisfactorily reproduces the complexity and mechanical parameters of facial tissues is not only challenging but also cost-intensive given its single-use predestination. The above enhances the competitiveness of the virtual analysis of stress distribution under external traumatic loads.

In the field of trauma surgery, FEA can be used to identify the regions of the craniofacial skeleton that are particularly prone to fractures [11]. It enables the precise mapping of stress distribution after trauma to the maxillofacial region, which can help in the evaluation of patients after a traumatic event. Additionally, a greater understanding of the biomechanics of traumas can optimize current surgical protocols for treatment of fractures.

So far, based on FEA, researchers have tested the influence of the presence of third molars on the weakness of the mandibular angle [12] (Table 1). They have also evaluated the possibility of iatrogenic fracture after removing various amounts of bone around impacted mandibular third molars, drawing the conclusion that if the surgical procedure involves the removal of the external oblique ridge, the stress occurring in the mandible under normal clenching is higher [13], which could allow oral surgeons to change their approach to tooth removal in certain cases. FEA of a traumatic load was also performed on a model of a mandible after the removal of a pathologic lesion. In this study, the theoretical efficacy of plate application for the decrease of stress on the mandible after surgical removal of a cyst was illustrated [14]. FEA of three different traumatic load conditions was also applied to the edentulous mandible which enabled the accurate mapping of stress distribution and the prediction of areas more susceptible to fracture among elderly people [15]. More complex traumas such as the isolated orbital floor fracture (IOFF) [16] or the zygomatic bone fracture [17], which are frequently observed in contact sports, were also studied by means of FEA and over the course of the studies their pathomechanisms were revealed.

As well as exploring the mechanisms of different fractures, FEA also meets researchers’ needs in analyzing relatively rare and more complex facial traumas, for example, to elucidate the damage to a human mandible in response to a blast event [18] or to conduct preliminary finite element simulation and analysis to determine the mechanism of mandibular damage in gunshot wounds [19].

The understanding of the pathomechanism of fractures is extremely important. It may find applications not only in industry (during designing helmets and other protectors for the head and neck) [20,21,22,23] and forensic medicine [24], but also in identifying the optimal bridging methods. The key factor for determining the long-term success of osseointegration is rigid fixation. The inappropriate selection of an osteosynthesis element may cause complications in bone fusion. Thus, there is a large interest in the FEA of different fixation methods and systems [25,26,27].

Sometimes, it can be troublesome not only to choose the fixation element but also to overcome the anatomical obstacles. Here, owing to its specific anatomy, a particularly challenging topic has always been condylar fracture osteosynthesis. Due to the demands of surgical access, the fixation element in this type of osteosynthesis should be exceptionally handy, easy to mount and durable in order to minimize the risk of consecutive intervention. Those considerations have been the object of numerous FEA studies [28,29,30]. Some research has investigated how the optimal stabilization of the condylar fracture fragments can be obtained [31,32], whereas another study, after a thorough evaluation by FEA, introduced a completely new type of osteosynthesis plate [33]. A tendency to introduce new, more optimized, more durable and more lightweight fixation elements leads to the assumption that there is still a vast field for FEA application in trauma surgery, in particular, when a movement towards materials which can undergo biodegradation can be seen [26]. New solutions and novel materials will always need thorough evaluation before launched in practice and so far FEA has proved to be a useful tool in such investigations.

## 4. Orthognathic Surgery

The selection of appropriate bridging elements is also a key determinant of successful outcomes in all orthognathic surgeries. In bilateral sagittal split osteotomy (BSSO), FEA has been eagerly used to compare the stability of bridging the bony segments with various fixation systems [34,35,36,37,38,39]. For this purpose, Stróżyk et al. created a three-dimensional digital isotropic model divided according to the BSSO line into three segments, with 5 mm gaps in between. The osteotomized fragments were bridged with three different fixation systems. Based on the displacement values for the osteotomized segments after a simulation of symmetrical biting with the incisors (20 N) and asymmetrical biting with the molars (80 N), the authors concluded that the most stable bridging after BSSO can be obtained with bicortical screw fixation [37], which stayed in line with another study of FEA where the use of 2.0 mm lag screws placed in a triangular configuration provided the most sufficient stability and fewer stress fields at the osteotomy site compared to other rigid fixation methods [40].

FEA reflecting a two-jaw osteotomy, a more complex surgery dedicated to patients with deformation of both the upper and lower jaws, was also conducted (Table 2). For example, Fujii et al. verified in a group of 50 patients qualifying for two-jaw osteotomy that FEA can be used to simulate pterygomaxillary disjunction during the LeFort I procedure not involving a curved osteotome (LF1-non-COSep) and to predict changes in the treatment outcome after extending the cutting line. Using preoperative CT image data, the authors created individualized digital models of the midface. Young’s modulus for individual voxels was calculated from the bone density determined during CBCT. Then, the virtual cut line was planned and the pterygomaxillary disjunction resulting from opening Tessier spreaders in the osteotomy fissure with a load of 150 N was simulated. Based on the predicted accumulation of maximal stress at the pterygomaxillary junction, the authors identified three types of disjunction. The rate of agreement between the FEA-predicted pterygomaxillary disjunction site and the actual disjunction site observed on postoperative CT images obtained 7 days after the LF1-non-COSep osteotomy with Tessier spreaders was 87%, which confirms that FEA has credibility in orthognathic surgery. Additionally, FEA demonstrated that the extension of the cutting line to the maxillary tuberosity was associated with a higher incidence of pterygoid process fractures [41], which is valuable information for clinicians.

Another orthognathic procedure performed frequently among patients with narrow maxillae is surgically assisted palatal expansion (SARPE). The distribution of tensions in maxillae that underwent SARPE was simulated by the finite element method and it was revealed that the steps in the zygomaticomaxillary buttress and the pterygomaxillary disjunction seem to be important to decrease the harmful dissipation of tensions during SARPE [42].

**Scheme 1:**
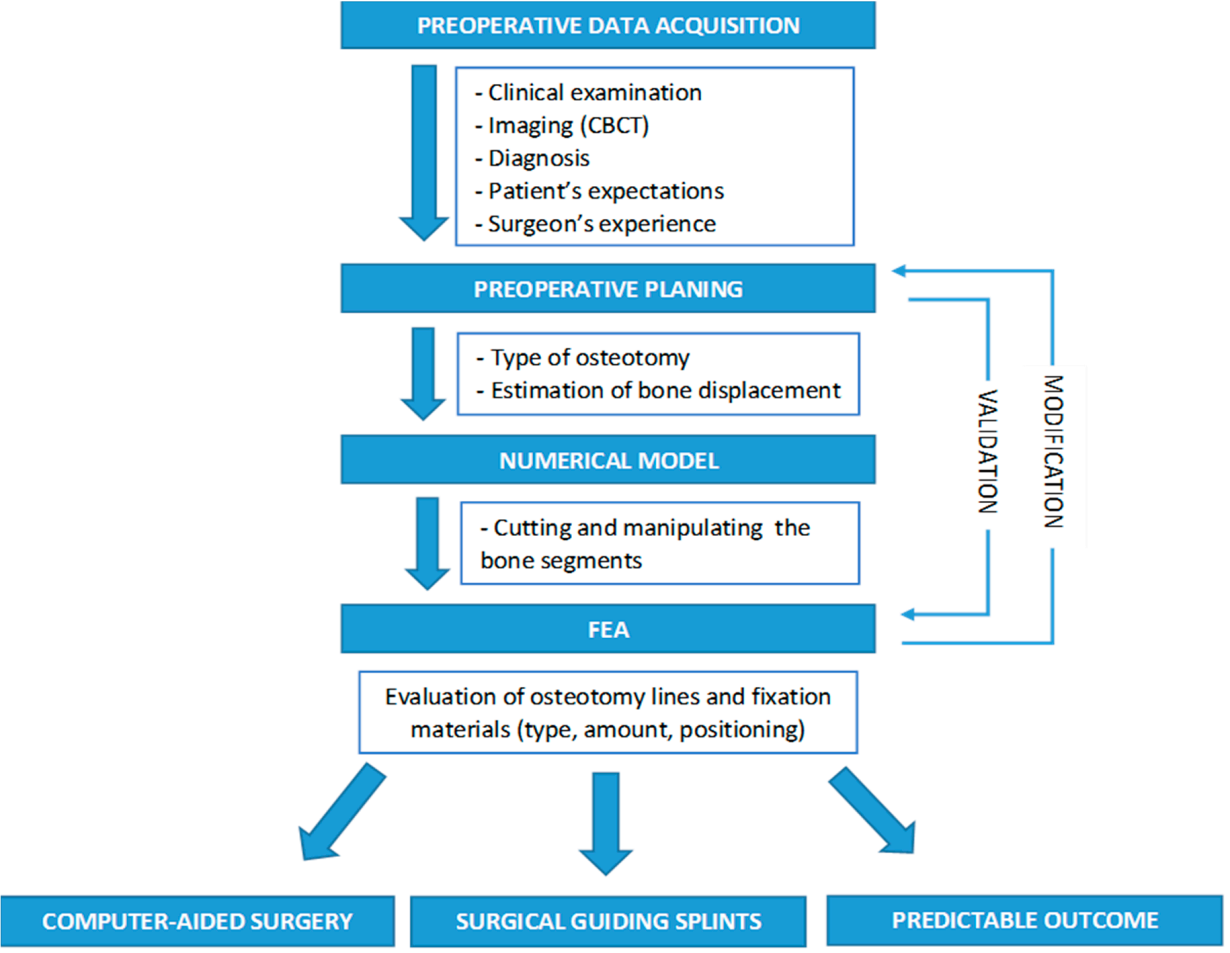
The application of FEA in computer-assisted orthognathic surgery planning.

In patients with large gnathic defects, even the preparation of a treatment protocol may be difficult and time-consuming. As a result, at many stages of treatment, its final effect may differ from the desired outcome. Therefore, Chabanas et al. proposed their own treatment protocol based on computer-assisted planning. The authors used FEA to simulate the facial consequences of scheduled orthognathic surgery [43]. Cevidanes et al. presented a computer-aided surgery system which included the construction of 3D models from the patient’s CBCT, dynamic cephalometry, semiautomatic mirroring, the interactive cutting of bone and the repositioning of bony segments for orthognathic surgeries, and indicated that FEA models are the most likely to provide a reliable simulation of facial soft-tissue changes resulting from skeletal reshaping [44]. Knoops et al. confirmed this thesis comparing the accuracy of soft tissue prediction between several available computer programs and a probabilistic finite element method (PFEM) in patients who underwent single-jaw Le Fort I maxillary advancement with vertical repositioning, and concluded that PFEM can indeed provide accurate soft tissue prediction and can therefore be useful at the time of preoperative patient communication [45]. Taking into consideration the possibilities that FEA offers not only with regard to soft but also to hard tissues, prospects for expanding the use of FEA in the future to implement more complex and individual solutions concerning the precise location of osteotomy lines and selection of the type, quantity and placement of fixation elements, to predict not only the visual but also the biomechanical outcome of scheduled othognathic surgery, can be expected (Scheme 1).

## 5. Reconstructive Surgery

FEA can be also used for the reconstruction of jawbones after extensive resections, usually due to oncological indications. From the viewpoint of postoperative management, the crucial parameters of the removed bone segment include its size, shape and location. Filling a bone defect within the maxilla or mandible should result in the restoration of its integrity and characteristic curvatures, and should create favorable conditions for further rehabilitation of the stomatognathic system. Using digital techniques, researchers can compare the level of stress at the bone-graft interface, to identify the type of transplant which is most suitable in a given clinical condition [46], and to find the most favorable conditions for appropriate bone fusion in the reconstructed region [47] (Table 3).

In reconstructive surgery, thorough and accurate preoperative planning with the involvement of the state-of-the-art virtual methods for modelling, simulation and analysis not only improves the outcome of the procedure and significantly shortens its duration, but also allows the operator to predict the behavior of the reconstructed region after exposure to various types of loads. Moreover, the standard elements for osteosynthesis available on the market may not provide an accurate reconstruction in more complex cases. In such cases, CT images from the patient may constitute the basis for an individual digital model of the reconstructed area, which is then used to design a tailor-made bridging element and to test its applicability by means of FEA [48]. For example, Moiduddin et al. created an integrated model to design and test a customized porous plate for the reconstruction of the mandible after the resection of its left body due to solid, plexiform-predominant ameloblastoma. The authors used preoperative CT datasets to create a virtual model of the mandible. Then, the tumor region was erased, reconstructed using a mirroring technique and used as a matrix to prepare a 2 mm thick reconstruction plate made of titanium alloy. The plate had multiple pores reducing its weight and allowing its fixation to the mandibular body and ramus with six bicortical screws. Using FEA for durability analysis, the authors demonstrated that the plate was resistant to the occlusal forces of the masseters, medial pterygoid and temporal muscles, and provided good stability for the reconstructed mandible [49].

When the correlation between bone structure and mechanical stresses was pointed out with the use of FEA, research on the influence of mechanical load on cell differentiation and tissue development flourished, especially in the field of skeletal tissue engineering scaffolds [52]. The influence of the different structural configurations and porosity of the scaffold on the distribution of stresses and strains was an issue in several FEA studies [50,51]. Hu et al. used CBCT images of a 50-year-old edentulous patient to reconstruct a 3D mandible and create an FEA model in which a segment of the mandibular body was virtually erased. The mandibular model was treated as an inhomogeneous material and the calculations of the elastic modulus of the bone were carried out in relation to bone mass density and Hounsfield units. Three types of porous scaffold grafts were designed to reconstruct the erased area. The grafts were subsequently fabricated with polylactic acid (PLA) material using a fused deposition modeling 3D printer. The ultimate load, yield load, failure deflection and yield deflection were compared both in physical model tests and finite element analysis. The results showed that the topological optimized graft provided the best mechanical properties. The authors also highlighted the great potential for numerical simulations and 3D printing technology in artificial porous graft design and manufacture [51].

## 6. Implantology

The correct assessment of bone conditions and ability to bear loads at implant anchoring sites is a determinant of successful outcome in implantoprosthetic treatment. An adequate balance between the implant, abutment and suprastructure can be obtained only under the ideal biomechanical conditions which directly influence remodeling of the bone. The appropriate height of the alveolar process can be maintained by the application of an optimally designed implant and a correctly modeled denture. Since FEA provides additional information about the distribution of stress at the surface of the implant and adjacent bone, it has become a basic instrument for designing intraosseous implants, and has found application in many previous implantoprosthetic studies [53,54,55,56]. FEA has been used to study not only implants anchored in the alveolar processes of the maxilla or the alveolar part of the mandible but also those found in other anatomical structures such as the zygomatic arch (Table 4). Ishak et al. compared the strength of anchorage of the zygomatic implants used as fixing elements for complete dentures in patients with atrophic maxilla, anchored using various surgical approaches. Using FEA, the authors determined that the level of stress in either the bone or the implant body was significantly higher if the denture was fixed with implants anchored using the intrasinus approach [57].

To satisfy the demand of implantologists, who frequently deal with the problem of insufficient amounts of bone for anchoring an intraosseous implant in patients with missing upper molars, digital models of the maxilla dedicated to stress and strain studies during the elevation of the maxillary sinus floor have been developed as well [58,59]. Using these, Schuller-Gotzburg et al. evaluated the influence of an augmented maxillary sinus lift with additional bone grafting. Over the course of the study it was established that stresses in compact bone were reduced significantly where a bone graft was used and that the reduction was most pronounced when the bone graft was placed in the lower third of the implant, closest to the sinus floor [60].

## 7. Limitations and Future Outlook

The domain of modernity is the fact that time has become a valuable product. Thus, experimental research that is detailed but not time-consuming, implementing predictable solutions which enable patients’ fast recovery, is typically praised. According to the reviewed literature, FEA is in this respect superior to most clinical and in vitro studies and has many advantages. It helps to formulate the right research question and design appropriate experiments. In complex systems where many variables need to be considered, it allows the manipulation of single parameters, making it possible to study the influence of each one, and provides almost instant insight into complex biomechanical intercourses which would be unable to obtain with any single clinical method. Exploiting the designed numerical model, the inflicted conditions can be freely modified and the study can be repeated as many times as desired. The patient is not exposed to potential risks associated with the application of novel materials or therapeutic procedures that have not been tested previously, and the need for animal experiments is eliminated. Surgeons have gained a tool to virtually perform osteotomies and bone relocations and to test the optimal positions, strains and stress of fixation materials in order to assess the best therapeutic materials and concepts. Scientists have obtained an instrument to optimize the design process in the product development cycle. The fact that virtual studies are associated with markedly lesser financial burden than conventional research is also worth emphasizing.

The visual interface of FEA stress and strain distribution is clear and easy to understand. It has definitely improved communication and cooperation between scientists and clinicians, encouraging them to pursue further research.

However, aside from its numerous advantages, FEA has its limitations too. It should be noted that the conditions of computerized studies are fully programmed by the researchers, and as such they will always pose a risk of bias. The most serious limitations of FEA are simplifications and assumptions. Early simulation models suffered from generalizations and a lack of geometric precision. Some more complex anatomical structures were commonly omitted at the bone modelling stage, which put the validity of the results into question. Another issue is the matter of reflecting not only the anatomy but also the morphology of tested tissues. In this area, simplifications were also commonly adopted. The authors of much research on FEA assumed that distinguishing between cortical and cancellous bone was irrelevant and considered the investigated bone, in general, as homogenous, isotropic and linearly elastic material [12,35,38]. Another disputed issue is the fact that the conducted analyses varied in terms of the material constants used (Table 5). For example, in the discussed literature, the adopted values of Young’s modulus for the cortical bone ranged from 13,500 to 18,000 MPa, whereas the Poisson’s ratio values for the abovementioned bone ranged from 0.22 to 0.3. There is no consensus on this matter. Not without significance is also the fact that at the moment, the greater the precision and accuracy in the geometric modelling of anatomical structures and morphological details, the more computational power is required, which makes the process time-consuming and more expensive. Moreover, some software currently available on the market predetermines the maximum level of numerical model discretization from the outset, rendering it impossible to conduct more detailed simulations [61].

The goal of future research should be to verify whether the assumptions mentioned above were not oversimplifications. To improve the quality of FEA-based research, particular attention should be paid to the stage of model creation [62]. At present, due to advancements in medical imaging based on scanners and computed tomography, obtaining a precise three-dimensional equivalent of a tested subject is accessible. Thanks to modern software tools capable of processing the obtained images and their compatibility with programs used later to run FEA, it has become within reach to generate accurate numerical models in reasonable time. Taking a closer look at the literature shows the tendency to create finer meshes of finite element models over the past few years. The model of a mandible published in 2009 by Oguz et al. consisted of 153,320 elements and 35,570 nodes [35], whereas six years later, Santos et al. reported results gained from a mandible model consisting of 528,010 elements and 759,787 nodes [15] (Table 6).

Along with the rapid progress in the implementation and market release of new technologies, the availability and decreasing costs of adequate software and hardware suitable for virtual analyses allowing a novel approach to solving some previously problematic aspects can be seen. In the matter of the evaluation of material constants, their individual calculation based on CT dataset has gained more enthusiasts. In the presented literature, for the calculation of Young’s modulus, Szucs et al., Tamura et al. and Fuji et al. converted each pixel of the CT dataset from Grey to Hounsfield units and using equations dedicated to this subject, calculated Young’s modulus from the obtained bone density. This approach enables researchers to distinguish stronger and weaker structures of the maxillofacial skeleton and as a result to create more realistic models [13,36,41]. When it comes to limitations in the numerical model discretization encountered as a result of software, attention has been directed towards the selective increase of the numerical model mesh density in the precise region of the investigated area [63].

To conclude, the thorough review of the provided literature indicates that FEA is a powerful tool in addressing many biomechanical problems and will undoubtedly continue to be applied in oral and maxillofacial research. Special attention should be placed on its wider implementation in research and practice in order to reduce the risk of unnecessary failure, extend knowledge of oral and maxillofacial biomechanics, introduce enhanced osteosynthesis solutions, reconstruction scaffolds, biomaterials or implant components and select the most optimal treatment materials and approaches.

## Figures and Tables

**Table 1:** Information regarding FEA conducted in the field of trauma surgery.

Author Year Reference	Anatomical Structure Modeled	Software Used	Study Design	Major Findings
Perestrelo (2016) [11]	Skull based on CT scan	InVesalius Rhinoceros Hypermesh	Simulation of a blowout fracture	Potentially weak areas to traumatic situation were revealed
Bezerra (2013) [12]	Mandibles with and without erupted third molars based on CT scans	ScanIP ANSYS 13	Simulation of a punch on the mandible	The mandibular angle becomes more fragile in the presence of third molars
Szücs (2010) [13]	Mandible with impacted third molars based on CBCT scan	Mimics ANSYS	Simulation of the extraction of the right impacted third molar with or without cortical bone removal	There is a possibility of mandible fracture during or after the extraction if large quantities of bone are removed
Santos (2014) [15]	Elderly edentulous mandible based on CT scan	InVesalius 3.0b Rhinoceros 5.0 ANSYS 14	Simulation of a traumatic load of 700 N applied to three different regions of the mandible	Potentially weak areas to traumatic situation were revealed
Wanyura (2011) [16]	Skull based on commercially available geometric 3D model	ANSYS 12.1	Simulations of isolated orbital floor fractures	Potentially weak areas to traumatic situation were revealed
Schaller (2011) [17]	Heads based on CT scans	Vworks 4.0 ANSYS 12	Simulation of a transient collision of two heads; results compared to a typical real patient case	Potentially weak areas to traumatic situation were revealed; the comparison with the real case revealed an identical fracture pattern
Levadnyi (2018) [23]	Head based on magnetic resonance images MRI	Mimics Abaqus 6.14	Investigation of a protective helmet effect in a simulation of the dynamic impact of a human head	A method to provide recommendations for protective helmet manufacturing was developed
Li (2019) [24]	3- and 4-month-old child heads based on CT scans	Hexotic LS-Dyna 971	Reconstruction of two suspected child abuse cases	The potential of FEA to explain the skull fracture patterns in the forensic investigation was demonstrated
Joshi (2018) [25]	Mandible based on CT scan	Geomagic ANSYS Workbench	Comparison of mono- and bicortical miniplate fixation in parasymphysis mandible fracture	The conclusion that both mono- and bicortical fixation provide sufficient lingual stability was reached
Park (2020) [26]	Mandible based on CBCT scan	SolidWorks Hypermesh Abaqus	Comparison of different fixation system materials at a unilateral mandibular fracture	The potential of biodegradable fixation materials was presented and the need for the preclinical evaluation of their efficacy was indicated
Joshi (2014) [27]	Mandible based on CT scan	Geomagic Unigraphics ANSYS Workbench	Comparison of different numbers, locations and design types of fixation with miniplates	The superior position of miniplates produced better stability than the inferior position; the number of screws did not affect fracture stability

**Table 2:** Information regarding FEA conducted in the field of orthognathic surgery.

Author Year Reference	Anatomical Structure Modeled	Software Used	Study Design	Major Findings
Stróżyk (2011) [37]	Mandible based on polyurethane model scan divided according to the bilateral sagittal split osteotomy (BSSO) line	-	Comparison of three fixation methods used in BSSO	Bicortical screw fixation demonstrates the best rigidity after BSSO
Gorashi (2019) [38]	Mandible based on CT scan divided according to the BSSO line	Mimics 17.0 Abaqus Geomagic 12.0 ANSYS	Comparison of the three most common fixation methods used in BSSO	Triangular screw fixation is better than one or two parallel plates
Hassan (2018) [39]	Mandible based on CT scan divided according to the BSSO line	Netfabb Rhinoceros Solidworks	Comparison of three fixation methods used in BSSO	The 1.7 mm miniplate has adequate strength to be used in BSSO, although it is less rigid when compared to the conventional 2.0 mm miniplate and 2.0 mm bi-cortical screws
Fuji (2017) [41]	Midface based on preoperative CT scan of patients diagnosed with prognathism	Mechanical Finder 6.2 OsiriX	Comparison of the pterygomaxillary dysjunction patterns predicted by FEA models with postoperative CT images and evaluation of extending the cutting line to predict the risk of pterygoid process fracture	FEA can be used to predict pterygomaxillary disjunction patterns during LFI-non-COSep and provides useful information for selecting safer procedures during LFI-non-COSep
De Assis (2014) [42]	Maxilla based on CT scan of a patient diagnosed with transverse maxillary deficiency	FEMAP 10.1.1 NEi Nastran	Comparison of four different types of surgically assisted palatal expansion (SARPE) osteotomies (with or without a step in zygomaticomaxillary buttress and with or without the pterygomaxillary disjunction)	Steps in the zygomaticomaxillary buttress and the pterygomaxillary disjunction decrease the harmful dissipation of tensions during SARPE
Chabanas (2002) [43]	Face based on preoperative CT scan	-	Presentation of a computer-aided maxillofacial sequence applied to othognathic surgery	The aesthetic surgical outcomes of bone repositioning can be studied with a biomechanical finite element soft tissue model
Knoops (2019) [45]	Face based on preoperative CT scan	Dolphin ProPlan CMF PFEM	Comparison of the prediction accuracy of four programs for 3D surgical planning	PFEM and ProPlan equally provide accurate soft tissue prediction and could be useful at the time of preoperative patient communication

**Table 3:** Information regarding FEA conducted in the field of reconstructive surgery.

Author Year Reference	Structures Modeled	Software Used	Study Design	Major Findings
Tie (2006) [46]	Mandibles reconstructed with different autogenous grafts based on CT scans of mandible, fibula and iliac crest	MedGraphics ANSYS 6.1	Investigation of the biomechanics of the mandible following reconstruction with autogenous bone grafts	Mandibles repaired with iliac crest grafts have more mechanical properties similar to normal than those repaired with fibula grafts
Cheng (2018) [47]	Edentulous mandibles with and without fibular graft based on CBCT scan	Mimics 16.0 3-matic 8.0 Geomagic 12 Abaqus 6.13	Evaluation of stress distribution and displacement of reconstructed and intact mandible under occlusal loading	The fibular graft placed at the intermediate location has the best biomechanics and provides favorable conditions for subsequent prosthetic reconstruction
Gutwald (2017) [48]	Mandible based on scan of synthetic model of mandible after resection and reconstruction	CADFEM ANSYS 15.0 OptiSLang	Comparison of the biomechanical performance of customized and standard mandibular reconstruction plates	Customized mandibular reconstruction plates have a better biomechanical performance than manually bent stock reconstruction plates
Moiduddin (2017) [49]	Mandible based on CT scan followed by virtual tumor resection and porous plate reconstruction	Mimics 17 3-Matic Geomagic ANSYS 17	Presentation of an integrated framework model for the design and analysis of a customized porous reconstruction plate	FEA reveals that the designed porous plate can withstand the chewing load conditions and provides good stability
Luo (2017) [50]	Mandible based on CT scan with virtual resection, titanium scaffold and bone graft material reconstruction	Mimics 10.01 Geomagic ANSYS 14.0	Designing and optimizing a three-dimensional tetrahedral titanium scaffold for the reconstruction of mandibular defects	Tetrahedral structural titanium scaffolds are feasible structures for repairing mandibular defects
Hu (2019) [51]	Edentulous mandible based on CBCT and with designed grafts	Mimics 16.0 Geomagic 12 Abaqus 6.13	Comparison of mechanical behavior of topological optimized grafts with round and square grafts	The topological optimized graft had the best mechanical properties

**Table 4:** Information regarding FEA conducted in the field of implantology.

Author Year Reference	Structures Modeled	Software Used	Study Design	Major Findings
Memari (2020) [53]	Computer-aided design (CAD)-simplified edentulous mandible and overdenture with bar and clip supported with two short or two long implants	Abaqus 6.1	Comparison of stress distribution around short (6 mm) and long (10 mm) implants in tow mandibular implant-supported overdentures	Using implants with different lengths in mandibular overdenture caused no major changes in stress distribution in peri-implant bone
Zhang (2016) [54]	Mandible based on CT scan and two types of CAD dental implants	Unigraphics NX 4.0 ANSYS Workbench 14.0	Evaluation of the stress distribution characteristics of 12 types of dental implants and surrounding bone with various abutments, implant threads and healing methods under different amounts of concentrated loading	A dental implant system characterized by a straight abutment, rectangle tooth and nonsubmerged healing may provide minimum value for the implant–bone interface
Wu (2019) [55]	Edentulous mandible based on synthetic jawbone model of CBCT and CAD implants, abutments and framework	Mimics 15.0 SolidWorks 2017 ANSYS Workbench	Evaluation of the all-on-four treatment with four osseointegrated implants in terms of the biomechanical effects of implant design and loading position on the implant and surrounding bone	For all-on-four treatment with four osseointegrated dental implants, altering the implant design does not appear to affect the biomechanical performance of the entire treatment
Lee (2019) [56]	CAD bone blocks and four types of implant components	3-Matic 9.0 Abaqus 6.14 FE-Safe 6.5	Evaluation of the stress and strain distribution of short implants and surrounding bone under loading conditions with four different connections	The abutment of the internal bone level showed the highest stress of the implant component
Ishak (2012) [57]	Maxilla and the zygomatic bone based on CT and CAD zygomatic implants and acrylic denture	Mimics 10.01 SolidWorks 2009 Abaqus	Comparison of two different types of surgical approaches, intrasinus and extramaxillary, for the placement of zygomatic implants in atrophic maxillae	The intrasinus approach demonstrates more satisfactory results
Schuller-Gotzburg (2018) [60]	Left maxilla based on CT scan and CAD bone graft and implant	3-Matic 5.1 SolidWorks 2010 ANSYS 13.0	Evaluation of the influence of an augmented sinus lift with additional inserted bone grafting	The low bone graft block position is associated with lower stress distribution in compact bone

**Table 5:** Models and material properties used in FEA of trauma to the maxillofacial region.

Reference	Model	Database	Young’s Modulus (MPa)		Poisson’s Ratio	
			Cortical Bone	Cancellous Bone	Cortical Bone	Cancellous Bone
Wanyura [16]	Skull	3D model	18,000	-	0.22	-
Santos [15]	Mandible	CT scan	13,700	1370	0.3	0.3
Schaller [17]	Head (detailed)	CT scan	*	-	0.326	-
	Head (simplified)	CT scan	13,500	-	0.32	-
Perestrelo [11]	Head	CT scan	13,700	-	0.35	-
Szucs [13]	Mandible	CBCT scan	*	*	0.3	0.3
Tie [46]	Mandible	CT scan	15,000	1500	0.33	0.3

* The radiographic density in the CT/CBCT scans was used to assign individual mechanical parameters.

**Table 6:** Differences between mandible models presented in FEA literature.

Reference	Properties of Bone	Number of Elements	Number of Nodes
Oguz (2009) [35]	Isotropic	153,320	35,570
Szucs (2010) [13]	Anisotropic	792,412	148,181
Santos (2015) [15]	Isotropic	528,010	759,787
Tamura (2018) [36]	Anisotropic	475,160	361,324
Ghorashi (2019) [38]	Isotropic	545,009	493,064
